# Delayed Recognition of a Rare Mediastinal Lymphoma Presenting as Postpartum Circulatory Collapse

**DOI:** 10.1155/2014/415352

**Published:** 2014-08-04

**Authors:** Jasmina Kevric, Sanil Nair, David Ernest

**Affiliations:** Intensive Care Unit, Monash Medical Centre and Monash University, 246 Clayton Road, Clayton, Melbourne, VIC 3168, Australia

## Abstract

We report a case of a 29-year-old primigravida at 36 weeks of gestation following an emergency caesarean section, complicated by respiratory distress and multiorgan failure secondary to superior vena cava (SVC) obstruction, requiring intubation and prolonged ventilatory support. The presented case highlights the consequences of delayed recognition of SVC obstruction due to a reluctance to undertake appropriate radiological imaging during pregnancy.

## 1. Case Report

A previously well 29-year-old primigravida at 36 weeks was transferred to our hospital following an emergency caesarian section for premature rupture of membranes. She had a preceding three-week history of dyspnoea, postural dizziness, headache, and neck and facial swelling for which specialist outpatient follow-up had been organized but not undertaken. No chest X-ray had been arranged due to fears of harmful effects given the patient's pregnancy state. A few hours following her uncomplicated caesarian section and delivery of healthy baby boy, she was noted to have worsening dyspnoea, respiratory rate of 44 breaths per minute, stridor, and engorged veins over her neck and chest and she was unable to lie supine. She was tachycardic (135 beats per minute) and normotensive, with an oxygen saturation of 94% on 8 L per minute of oxygen. She remained conscious throughout the deterioration.

**Figure 1:**
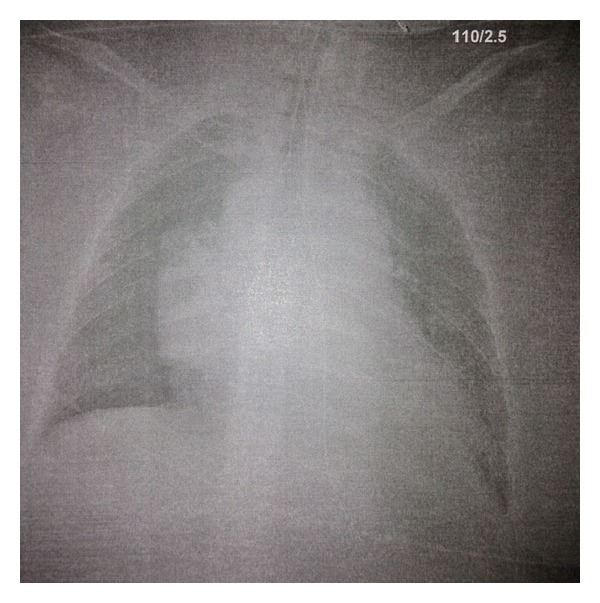
Chest X-ray showing large mediastinal mass.

Investigations at that time revealed a normal serum haemoglobin (123 g/L; RR: 100–170 g/L), normal white cell count (11.1; RR: 4.0–15.0 10^9^/L), normal platelet count (276; RR: 150–500 10^9^/L), an elevated INR (1.7; RR 0.8–1.2), renal dysfunction (urea 12.2 mmol/L; RR: 2.5–7.0 mmol/L; creatinine 195 umol/L; RR: 30–90 umol/L) from a baseline creatinine level 58 umol/L, and liver dysfunction (ALP 237 (RR: 20–120 U/L), GGT 64 U/L (RR: <30 U/L) and ALT 290 U/L (RR: <40 U/L), and total bilirubin 18 umol/L (RR: 1–20 umol/L)), and a chest X-ray revealed a large mediastinal mass (Figure 1). The patient was initially managed with diuretics, bronchodilators, and intravenous dexamethasone (total of 8 mg).

Given the clinical setting, an emergency intubation was undertaken by senior anaesthetists in the operating theatre using a combination of propofol and ketamine, suxamethonium (100 mg), and aramine (1 mg). Immediately following intubation, she developed circulatory collapse and was managed using intravenous adrenaline boluses (10 mg in total) followed by an adrenaline infusion and atracurium (45 mg total). She was commenced on an intravenous infusion of morphine and midazolam and transferred to our centre for ongoing management in an intensive care unit (ICU).

At the time of her admission to ICU, 12 hours following delivery, the patient was noted to be tachypnoeic (respiratory rate 44 breaths/min), bradycardic (pulse 30 beats/min), and markedly hypotensive, for which she was further resuscitated. An arterial blood gas analysis revealed pH 6.89 (RR: 7.35–7.45), pCO2 28 mmHg (RR: 36–44 mmHg), pO2 70 mmHg (on 50% of FiO2), bicarbonate 5 mmol/L (RR: 22–32 mmol/L), lactate 20 mmol/L (RR: 0.5–2.0 mmol/L), and haemoglobin 90 g/L (RR: 120–160 mmol/L), indicative of a severe metabolic acidosis and hypoxaemia. A chest X-ray and subsequent CT chest scan showed a large mediastinal mass measuring 7 cm × 10 cm × 10 cm invading and occluding the superior vena cava, extending onto the right atrium and displacing the ascending aorta. Further investigations included a liver ultrasound, which revealed portal vein thrombosis with extension into the splenic vein, and a transthoracic echocardiography study, which revealed a small pericardial effusion and moderate to severe reduction in overall systolic function. The patient was managed with mechanical ventilation, inotropic support, renal replacement therapy, and anticoagulation for portal vein thrombosis.

On day 3, a CT guided biopsy of the mediastinal mass revealed a high grade B cell lymphoma (CD 20 and CD 30 positive), and a bone marrow biopsy showed evidence of dyserythropoiesis, for which the patient was treated with combination chemotherapy R-CEOP (rituximab, cyclophosphamide, etoposide, vincristine, and prednisolone) resulting in small reduction of tumour mass observed on day 20. The patient was uneventfully extubated after 25 days and discharged on day 27 for further management of her lymphoma.

## 2. Discussion

Superior vena cava (SVC) obstruction secondary to a mediastinal mass is an uncommon presentation and more typically associated with lung and breast neoplasms than lymphomas. SVC obstruction secondary to neoplasms usually develops gradually and symptoms may include dyspnoea, particularly in the supine position, neck swelling, neck vein distension, and cough.

The diagnosis of SVC obstruction in pregnancy is confounded by the similarity of the initial clinical features to normal physiologic responses in pregnancy and a potential reluctance to undertake appropriate radiological imaging, as in our case. Nevertheless, unexplained and prolonged dyspnoea should alert the clinicians to seek alternative causes in pregnant women.

The use of radiology in pregnancy was a significant pitfall in this case as the treating physicians were reluctant to expose the fetus to radiation. Ionising radiation from X-rays consists of high-energy photons which, at high doses, can lead to DNA damage and formation of free radicals, with 10 to 17 weeks of gestation considered to be the most sensitive period for central nervous system teratogenesis [1]. The American Academy of Pediatrics and the American College of Obstetricians and Gynaecologists have recommended that “diagnostic radiologic procedures should not be performed during pregnancy unless information obtained from them is necessary for the care of the patient and cannot be obtained by other means” and further that women should be counselled that exposure to less than 5 rad has not been associated with fetal anomalies or pregnancy loss and that exposure to single X-ray (0.00007 rad) does not result in harmful fetal effects. Given these recommendations, it would have been reasonable to perform necessary diagnostic imaging at the time of our patient's presentation with unexplained dyspnea.

This case further highlights that a patient with a suspected mediastinal mass requiring intubation is at risk of developing circulatory collapse. Contributing factors include the supine position, associated with reduced venous return due to inferior vena cava compression, and the use of general anaesthesia and paralytic agents, and cardiorespiratory instability may result from tracheobronchial tree obstruction, superior vena cava obstruction, and compression of heart and pulmonary artery [2]. Difficulties associated with the intubation should be anticipated in such cases and adjuvants, such as fibreoptic scopes, should be readily available. Optimal positioning is required to prevent further compression of the trachea, inhalational agents, such as sevoflurane, and intravenous agents, such as etomidate, with minimal effects on the haemodynamics should be preferred, and full invasive blood pressure monitoring is advisable [3]. Steroids may be useful to decrease airway oedema and have been shown to reduce the inflammatory response associated with tumour invasion [4].

Our patient was diagnosed with high grade B cell lymphoma, a form of non-Hodgkins lymphoma, which in most pregnant women is an aggressive histologic subtype [5]. The case illustrates the importance of investigating unexplained symptoms, such as dyspnoea, in pregnancy and highlights the consequences of delayed diagnosis of SVC obstruction due to a reluctance to undertake appropriate radiological imaging during pregnancy and the need to anticipate challenges associated with endotracheal intubation.
